# A Prospective Controlled Study on the Longitudinal Effects of Rehabilitation in Older Women with Primary Sarcopenia

**DOI:** 10.3390/life15040609

**Published:** 2025-04-06

**Authors:** Bianca Maria Vladutu, Daniela Matei, Anca Maria Amzolini, Constantin Kamal, Magdalena Rodica Traistaru

**Affiliations:** 1 Doctoral School, University of Medicine and Pharmacy of Craiova, 200349 Craiova, Romania; biancamariavld@gmail.com; 2 Department of Medical Rehabilitation, University of Medicine and Pharmacy of Craiova, 200349 Craiova, Romania; rodica.traistaru@umfcv.ro; 3 Department of Medical Semiology, University of Medicine and Pharmacy Craiova, 200349 Craiova, Romania; 4 Department of Family Medicine, University of Medicine and Pharmacy of Craiova, 200349 Craiova, Romania; constantin.kamal@umfcv.ro

**Keywords:** sarcopenia, physical performance, health-related quality of life, personalized rehabilitation, older adults, SarQoL, SPPB

## Abstract

Sarcopenia, defined as a progressive loss of skeletal muscle mass, strength, and function, is a leading contributor to disability, dependence, and reduced quality of life (HRQoL) in older adults. This study aimed to evaluate the impact of a personalized six-month rehabilitation program, centered on tailored kinetic therapy, on physical performance and HRQoL in older women with primary sarcopenia. Methods: This prospective controlled study included 80 women aged ≥65 years, allocated into a Study Group (SG, *n* = 40), who followed a supervised personalized kinetic program, and a control group (CG, *n* = 40), who received general advice regarding physical activity and nutrition. Physical performance was measured using the short physical performance battery (SPPB), while HRQoL was assessed with the disease-specific SarQoL questionnaire. Evaluations were conducted at baseline and after six months. Results: At baseline, both groups had comparable scores (SPPB: SG = 5.75 ± 0.86 vs. CG = 5.8 ± 0.88, *p* = 0.798; SarQoL: SG = 54.42 ± 8.76 vs. CG = 55.59 ± 4.61, *p* = 0.457). After six months, the SG showed significant improvements (SPPB = 8.05 ± 0.90, *p* < 0.001; SarQoL = 62.55 ± 7.00, *p* < 0.001). Significant gains were observed in domains related to physical and mental health, locomotion, functionality, and leisure activities (*p* < 0.05). In contrast, the CG showed only minor, non-significant changes (SPPB = 6.17 ± 0.78; SarQoL = 56.51 ± 5.51). Conclusions: A structured, personalized kinetic program significantly improves physical performance and HRQoL in older women with primary sarcopenia. These results support the need for individualized, supervised rehabilitation programs in optimizing functional recovery and enhancing patient-centered outcomes in sarcopenia management.

## 1. Introduction

The remarkable increase in life expectancy, combined with declining fertility rates, has led to a rapidly aging global population. By 2050, the population aged 60 years and older is expected to exceed two billion, representing one of the most profound demographic shifts in modern history [1]. While this trend reflects advances in public health and medicine, it also raises significant challenges related to preserving the health, independence, and quality of life of older adults [2].

Among the age-related conditions affecting this population, sarcopenia—the progressive loss of skeletal muscle mass, strength, and function—holds particular significance [3]. The decline in muscle mass progresses from approximately 50% of total body weight in young adults to around 25% after the age of 80 [4]. This process accelerates in the presence of chronic diseases, poor nutrition, and physical inactivity [1,5], amplifying functional impairment and reducing quality of life.

Diagnostic criteria for sarcopenia have evolved over time, largely shaped by the contributions of the European Working Group on Sarcopenia in Older People (EWGSOP2) and the Asian Working Group for Sarcopenia (AWGS 2019) [6], with EWGSOP2 defining it as a progressive, generalized skeletal muscle disorder primarily identified by reduced muscle strength, further confirmed through low muscle mass and impaired physical performance [7].

Sarcopenia was officially recognized as a disease in 2016, when it was assigned the ICD-10-CM code M62.84, prompting increased clinical and scientific attention [3,7]. The condition is classified into the following: primary sarcopenia, related solely to aging [8], and secondary sarcopenia, linked to factors such as chronic illnesses (osteoporosis, cardiovascular disease, diabetes, long COVID syndrome), poor nutrition, or low physical activity levels [9,10,11]. Prevalence varies widely, ranging from 5% to 29% in community-dwelling older adults and reaching up to 50% in hospitalized or institutionalized individuals [12]. Sarcopenia is more common in women due to lower baseline muscle mass and hormonal differences [1,13].

The onset and progression of this condition result from a combination of complex biological, environmental, and lifestyle factors. Central to its pathogenesis is the disruption of muscle protein turnover, resulting from an imbalance between synthesis and degradation [13,14,15,16,17]. This imbalance is further aggravated by age-related anabolic resistance, declining levels of anabolic hormones such as testosterone, estrogen, and growth hormone [18], chronic low-grade inflammation (“inflammaging”) [19], mitochondrial dysfunction, and oxidative stress [20]. Together, these mechanisms create a progressive cycle of muscle degradation, functional decline, and physical frailty [21].

The SARC-F questionnaire serves as a practical and accessible screening tool, relying on self-reported difficulties in five key areas: muscle strength, walking, rising from a chair, climbing stairs, and history of falls. A score of 4 or higher indicates a probable diagnosis and signals the need for further diagnostic assessment [22,23]. A comprehensive evaluation of muscle health and functional capacity requires a multimodal assessment, combining measurements of:

- Muscle strength, typically assessed through handgrip strength or the chair stand test [6,24];
- Muscle mass, evaluated using bioelectrical impedance analysis (BIA) or dual-energy X-ray absorptiometry (DXA) [24];
- Physical performance, measured by gait speed, the short physical performance battery (SPPB), or the Timed Up and Go (TUG) test [6,24];
- In certain cases, advanced imaging techniques, such as Magnetic Resonance Imaging (MRI) and Computed Tomography (CT), provide highly accurate data on muscle composition and quality. However, these methods are less practical for routine use due to their high costs and specialized equipment requirements [25].

Among the available tools, BIA has emerged as a practical and cost-effective option, particularly suitable for outpatient settings, with results correlating well with those obtained from MRI [26,27].

Beyond its physical consequences, sarcopenia significantly affects health-related quality of life (HRQoL), influencing not only self-perceived physical capacity, as assessed by HRQoL tools, but also psychological well-being and social participation [28]. To assess these complex impacts, the sarcopenia quality of life (SarQoL) questionnaire was specifically developed and validated as a disease-specific instrument [29]. The SarQoL evaluates seven key domains (physical health, locomotion, body composition, functionality, activities of daily living, leisure activities, psychological well-being). Although the SarQoL has been extensively used in cross-sectional studies, evidence remains scarce regarding how its scores evolve following structured rehabilitation programs specifically designed for sarcopenic patients. Investigating these changes is essential for optimizing rehabilitation strategies and developing patient-centered interventions that directly target the domains most affected by the disease [29].

Given its multifactorial nature, sarcopenia requires a multimodal therapeutic approach, combining the following:

- A kinesiotherapy program, proven to improve muscle strength, mass, and physical performance [30]. Numerous studies confirm the benefits of individualized resistance and aerobic training in combating sarcopenia-related muscle atrophy. Resistance training using elastic bands or bodyweight improves type II fiber recruitment and functional mobility, while aerobic and balance exercises enhance coordination and reduce fall risk [24,31,32].
- Nutritional interventions, ensuring optimal protein intake and correcting frequent vitamin D deficiency in older adults [33]. Combining exercise with protein supplementation, which shows superior results compared to either intervention alone [24]; Adequate protein intake (1.2–1.5 g/kg/day) is essential for maximizing muscle protein synthesis, particularly when synchronized with post-exercise recovery windows. Meta-analyses have demonstrated synergistic effects between resistance training and leucine-rich protein supplementation in improving muscle function in older adults [34,35].
- Comorbidities management, particularly addressing metabolic imbalances such as insulin resistance and dyslipidemia, which further accelerate muscle loss [36]. Interventions targeting glycemic control and lipid balance can indirectly support muscle preservation. Recent evidence highlights the role of insulin sensitivity in maintaining anabolic signaling in skeletal muscle, underscoring the importance of systemic metabolic regulation [36].

Adjunctive modalities, such as deep oscillation therapy, may enhance the effects of kinesiotherapy. Deep oscillation via manual applicator delivers low-frequency electrostatic impulses that penetrate soft tissues, generating mechanical vibrations. These oscillations improve local circulation, reduce muscle tension, and stimulate lymphatic drainage, creating optimal preconditions for movement therapy [37,38,39,40].

Despite significant progress in understanding sarcopenia, there is still limited evidence regarding the effectiveness of personalized kinetic programs tailored to individual functional deficits and rehabilitation goals. Such programs could better address patients’ specific needs, leading to more meaningful improvements in both physical performance and quality of life.

This study introduces a personalized rehabilitation protocol that combines targeted physical exercises with individualized progression plans. By filling this evidence gap, the study aims to provide actionable clinical insights and contribute to developing more patient-centered sarcopenia management strategies.

Objective: To evaluate the effectiveness of a personalized kinetic program in improving physical performance and HRQoL in older adults with primary sarcopenia.

By exploring these relationships, this study aims to enhance scientific understanding of tailored rehabilitation and support the clinical implementation of individualized rehabilitation programs, with the ultimate goal of improving functional capacity, preserving independence, and enhancing quality of life in older adults with sarcopenia.

## 2. Materials and Methods

### 2.1. Design Overview

This prospective controlled interventional study was conducted between October 2023 and June 2024 in the Department of Physical Medicine and Rehabilitation at Filantropia Hospital, Craiova. Group allocation was based on participants’ willingness to engage in additional physical activity, reflecting real-world clinical practice. Although participants were not randomly assigned, baseline characteristics between groups were comparable, and potential confounding factors were controlled for through statistical adjustments to minimize selection bias and enhance the study’s internal validity.

Participant Selection and Screening: A total of 300 consecutive elderly patients admitted to the department were initially screened for sarcopenia using the SARC-F questionnaire (strength, ambulation, rising from a chair, stair climbing, and history of falling questionnaire), a validated tool for identifying individuals at risk of sarcopenia. Patients with a SARC-F score > 4 (*n* = 140) underwent further clinical, laboratory, and functional evaluations, including SPPB to evaluate physical performance and SarQoL questionnaire to assess quality of life related to sarcopenia.

To rule out secondary or acute sarcopenia, a comprehensive laboratory workup was performed, including inflammatory markers, metabolic profiles, and other relevant tests.

Final Sample: Following the initial screening and comprehensive evaluation, a total of 96 patients met the diagnostic criteria for severe primary sarcopenia, as defined by the EWGSOP2 [24]. All eligible individuals during the recruitment period were women. The final sample was established based on strict inclusion and exclusion criteria, full compliance with the screening protocol (including SARC-F, SPPB, and SarQoL assessments), and voluntary consent to participate in the study. Patients were evaluated at baseline (T1) by a multidisciplinary team comprising physical medicine specialists and physiotherapists.

Participants were divided into two groups:

- Study Group (SG): A total of 50 patients enrolled in a structured, individualized rehabilitation program;
- Control Group (CG): A total of 46 patients who maintained their usual daily activities without additional physical interventions.

The SG participated in a 6-month personalized rehabilitation program designed to fit their compliance levels and integrate into their daily routines. The program included resistance exercises, balance training, and flexibility exercises, with periodic supervision to ensure adherence. The CG continued with their regular daily activities without any supervised physical training.

A follow-up assessment was conducted at 6 months (T2) to evaluate the long-term effects of the intervention. Parameters assessed included physical performance (SPPB) and quality of life (SarQoL).

For statistical validity, only participants who achieved the following were included:

- Completed both T1 and T2 evaluations;
- Attended at least 80% of the rehabilitation sessions (for SG);
- Provided complete and valid data.

**Figure 1:**
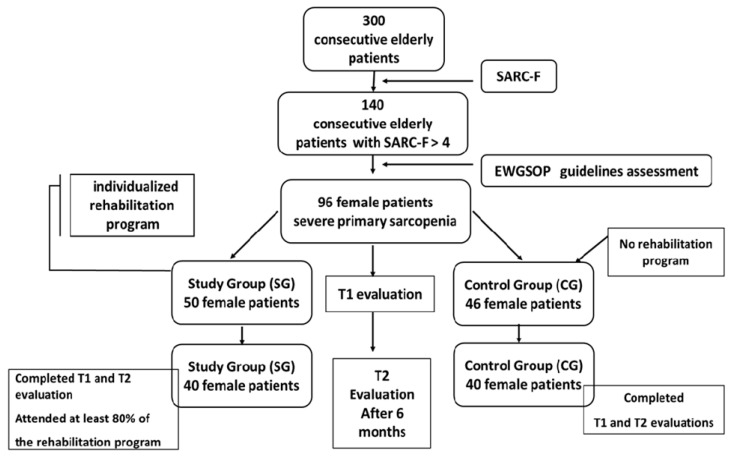
Diagram of study (EWGSOP = European Working Group on Sarcopenia in Older People, SARC-F questionnaire).

Eighty patients were included in the final analysis. In the SG, 40 patients (80%) completed the program and follow-up assessments, while 40 patients (86%) in the CG completed the follow-up (Figure 1).

### 2.2. Participants

Eligible patients were identified based on predetermined inclusion and exclusion criteria.

Inclusion Criteria: Participants were considered eligible if they met the following conditions: (a) aged ≥ 65 years [30]; (b) BMI between 24 and 30 kg/m^2^; (c) diagnosis of sarcopenia according to the criteria established by the European Working Group on Sarcopenia in Older People 2 (EWGSOP2) [24], the 2019 Asian Working Group for Sarcopenia (AWGS) [41], and the Sarcopenia Definition and Outcomes Consortium (SDOC) [42]; (d) stable comorbid conditions: mild to moderate osteoarthritic conditions (hip, knee, spine) [43,44], hip or knee arthroplasty older than 1 year, osteoporosis under treatment, with no fractures in the past year, therapeutically controlled primary hypertension (BP < 140/90 mmHg), well-controlled type 2 diabetes (HbA1c < 7.5%); (e) no musculoskeletal injuries within the last 6 months; (f) stable neuropsychological status without cognitive impairments; (g) no participation in a formal rehabilitation program within the last 12 months; (h) self-reported health status confirmed through a structured questionnaire (SARC-F); (i) for the CG, willingness to maintain usual daily activities and attend follow-up evaluations; and (j) willingness to participate in the rehabilitation program (for the SG).

Exclusion Criteria: Participants were excluded if they presented factors beyond the natural aging process, including factors that could interfere with sarcopenia-related outcomes: (a) dependent living situations; (b) diagnosed psychiatric disorders confirmed by a mental health specialist; (c) recent traumatic injuries, burns, or prolonged immobilization; (d) presence of severe comorbidities, such as malignancies, neurological or hematological disorders, heart failure, severe chronic obstructive pulmonary disease (COPD), severe infections; (e) organ failure of any etiology; (f) acute severe illnesses at the time of enrolment; (g) diagnosed frailty syndrome, sarcopenic obesity, or malnutrition-associated sarcopenia; (h) chronic use of medications known to influence muscle mass or function, including: corticosteroids, opiates, antibiotics, non-steroidal anti-inflammatory drugs (NSAIDs), digoxin, metformin, anticholinergics, iron, potassium supplements, proton pump inhibitors, antacids, sedatives, neuroleptics, and hormonal therapies; (i) compulsive eating behaviors or diagnosed eating disorders, (j) acute COVID-19 infection or post-COVID-19 syndrome with functional impairment; (k) hospitalization for any reason within the past 3 months; (l) severe uncorrected hearing or vision impairments that would interfere with participation in the rehabilitation program; (m) physician-recommended activity restrictions; and (n) lack of compliance or unwillingness to participate in the rehabilitation program (applicable to participants in the Study Group).

### 2.3. Study Intervention

This study implemented a structured, patient-centered rehabilitation program for the Study Group (SG), designed to improve physical performance and quality of life in elderly patients with severe primary sarcopenia. The intervention was carried out under the supervision of specialized physiotherapists in the Department of Physical Medicine and Rehabilitation at Filantropia Hospital, Craiova.

Program Design and Implementation:

The rehabilitation program was conducted in three distinct stages to ensure gradual progression and sustained patient engagement:

- Stage 1 (Supervised Initiation)—In-hospital kinesiotherapy and deep oscillation therapy (2 weeks): Twelve supervised sessions of kinesiotherapy combined with deep oscillation therapy. Sessions were conducted daily under the guidance of a specialized physiotherapist to ensure adherence, safety, and correct exercise execution. This phase emphasized comprehensive monitoring and individualized attention to foster behavior changes and maximized the program’s effectiveness. The detailed program is included in Table 1;
- Stage 2 (Home-based Maintenance)—Home-Based Kinetic Training Program (5 months): Patients followed a personalized home exercise plan with monthly outpatient evaluations to monitor progress. Weekly telephone follow-ups were conducted by the physiotherapist to ensure compliance, provide motivation, and address potential barriers. Patients received detailed educational brochures outlining daily exercises to facilitate adherence. The detailed program is included in Table 2;
- Stage 3 (Reinforcement and Optimization)—Outpatient Kinesiotherapy and deep oscillation therapy (2 weeks): Resumption of 12 kinesiotherapy and deep oscillation sessions at the outpatient clinic. This phase reinforced exercise habits and optimized functional gains achieved during the home-training phase. The detailed program is included in Table 1.

Intervention Details:

- Kinesiotherapy Program: The objectives of the applied kinesiotherapy program were to improve muscle strength, balance, flexibility, and endurance, thereby reducing sarcopenia-related functional decline. The kinesiotherapy sessions focused on the following components: flexibility exercises (to improve joint mobility and reduce stiffness, enhancing muscle elasticity and preventing injuries); resistance (strength) training (targeting agonist-antagonist muscle groups using resistance bands, small weights, and bodyweight exercises); balance and gait training (implementing proprioceptive exercises on unstable platforms to improve coordination and reduce fall risk); aerobic endurance training (low-intensity activities (e.g., walking, cycling) performed at 40–60% of the maximum heart rate, based on the formula: (220 − age) × 0.65); functional training (mimicking daily activities like stair climbing, sit-to-stand transitions, and carrying light objects to enhance daily living skills);
- Deep oscillation therapy was utilized to prepare skeletal muscles for the kinesiotherapy program, enhance local metabolism, reduce muscle stiffness, and improve muscle function [37,38,39,40]. A personal device (DOP1.1.—INDIVID, PHYSIOMED ELECTROMEDIZIN A.G., Germany, Series 2442007) was used, applying high-frequency oscillations (100 Hz) for analgesic and muscle-relaxing effects, followed by low-frequency oscillations (5–25 Hz) to stimulate metabolic activity.

To complement the intervention, all participants (SG and CG) received the following:

- Nutritional recommendations: targeting 1.2–1.5 g/kg of protein daily to maximize muscle protein synthesis [34];
- Educational support: counseling on sarcopenia awareness, the importance of physical activity, and long-term self-management strategies.

### 2.4. Parameters and Measurements

The assessment of sarcopenia in this study was conducted using a comprehensive set of validated clinical, functional, and quality of life measures to ensure accuracy and methodological rigor. This multidimensional approach enhances the reliability of the findings and supports the study’s objectives.

- The SARC-F Questionnaire was used as a screening tool. It includes five components: strength, assistance with walking, rising from a chair, stair climbing, and a history of falling. Each component is scored from 0 to 2 points, with a total score ranging from 0 to 10. A score of ≥4 is predictive of sarcopenia and indicates the need for further diagnostic evaluation [23];
- Muscle Strength Assessment: HGS was measured using the Saehan Squeeze Dynamometer (Baseline 12-0290 Dynamometer, Pneumatic Squeeze Bulb 30 30 PSI, without Reset Fabrication Enterprises (FEI)–USA, Importer, Romania), a validated tool strongly correlated with lower-extremity strength. Participants stood upright with the dynamometer beside them, but not touching their bodies. They performed three maximal isometric contractions of 5 s each with their dominant hand, with 60 s rest intervals between trials. The highest value was recorded for analysis. Cut-off values: <27 kg for men and <16 kg for women, as recommended by the European Working Group on Sarcopenia in Older People (EWGSOP2) [24];
- Anthropometric Measurements: Height (m), body mass (kg), BMI, (kg/m^2^), and mid-upper arm circumference (MUAC) were performed. MUAC was measured at the midpoint between the olecranon and acromion with the elbow flexed at 90°. A cut-off value of <22.5 cm indicates reduced muscle reserves [25]. All anthropometric assessments were conducted following standardized procedures to ensure reliability and reproducibility across evaluations.
- Body Composition Analysis
  1. Bioelectrical impedance analysis (BIA) estimates body composition by measuring the impedance of a low-frequency electrical current passed through the body. Since fat and muscle conduct electricity differently, this technique effectively monitors changes in body composition over time. Standardization: To minimize variability, BIA measurements were performed under controlled conditions—at the same time of day, after fasting, and following a period of rest. The same evaluator conducted all measurements to reduce inter-observer variability [26]. BIA offers an optimal balance between affordability and accuracy, making it suitable for routine clinical practice. The EWGSOP2 recommends BIA as a valid method for assessing muscle mass in clinical settings [27]. BIA is recognized in guidelines as a valid approach for detecting and screening sarcopenia. It offers a non-invasive, cost-effective, and portable solution for assessing body composition with reliability [45];
  2. Skeletal Muscle Mass (SMM): Measured using the Omron Healthcare BIA device (Body Fat Monitor, BF511, Omron 4015672104051, Global headquarters: Kyoto, Japan, Imported/distributed by MedTehnica, Romania). A pathological SMM was defined as values below 24% of the patient’s total body weight;
  3. The Skeletal Muscle Mass Index (SMMI) was calculated as appendicular skeletal muscle mass divided by height squared (kg/m^2^). The cut-off value for sarcopenia in women was <5.7 kg/m^2^, as per EWGSOP2 guidelines [24];
  4. Skeletal Muscle Percentage (SM%) represents the proportion of an individual’s total body weight that is composed of SMM. It provides a useful metric to assess muscle health relative to body size, offering insights into body composition beyond absolute muscle mass measurements [46]. While SM% is not always included as a diagnostic criterion for sarcopenia, it can complement other metrics like SMM and SMMI to provide a more comprehensive assessment of muscle status.
- The physical performance assessment was used to establish sarcopenia severity and ensure a homogeneous study cohort. Physical performance was evaluated using the following standardized tests:
  1. Timed Up-and-Go (TUG) Test: Participants rose from a chair without using their arms, walked 3 m, turned, and returned to the chair. The fastest time of the three trials was recorded. According to reviewed studies, the Timed Up and Go test is clinically relevant and demonstrates reliability across various groups. Its broad applicability in clinical settings makes it a versatile tool for selecting activity-based outcome measures. Particularly in geriatric assessments, the Timed Up and Go test is extensively researched and utilized [47];
  2. Gait Speed Calculation: (6/TUG time) × 1.62. Gait speed ≤ 0.8 m/s indicates impaired mobility and is associated with an increased risk of adverse health outcomes [48];
  3. The short physical performance battery (SPPB) assessed lower-limb function through three components: the balance test (participants maintained three different positions for 10 s each: feet together; semi-tandem stance; tandem stance: heel-to-toe); chair stand test, which assessed lower-limb strength by timing the participant rising from a seated position without using their arms; the 4-meter gait speed test (measures walking speed over a short distance. Each subtest is scored from 0 to 4 points, with a maximum total score of 12. A score ≤8 indicates severe sarcopenia [49,50]. SPPB is a valid and reliable tool for use with older patients and is recommended as part of the comprehensive geriatric assessment for the evaluation of the physical or functional status [51].
- A quality of life assessment was performed using the SarQoL questionnaire, a disease-specific, patient-reported outcome measure designed to assess health-related quality of life (HRQoL) in individuals with sarcopenia. It consists of 55 items integrated into 22 questions covering seven domains: D1 (physical and mental health), D2 (locomotion), D3 (body composition), D4 (functionality), D5 (activities of daily living), D6 (leisure activities), D7 (fears). Most items use a Likert scale to assess frequency or intensity, with responses scored from 0 to 100. A higher score indicates better HRQoL [29]. The sarcopenia quality of life^®^ (SarQol^®^) questionnaire is a specific tool designed for evaluating quality of life (QoL) in sarcopenia. Its adaptation across different languages and cultural contexts has confirmed its validity and reliability for assessing QoL in elderly sarcopenic patients [52]. The Romanian version of the SarQoL questionnaire was administered to all participants. Scoring followed the official algorithm provided by the questionnaire developers, available online [52];
- Laboratory Evaluations: To exclude secondary causes of sarcopenia and control for potential confounders, comprehensive laboratory testing was performed, including standard biochemical markers (C-reactive protein, fibrinogen, lipid profile) and inflammatory biomarkers (adiponectin, leptin, and tumor necrosis factor-alpha). These markers were measured using commercially available ELISA kits (Biovendor R&D, Brno, Czech Republic), following standardized laboratory protocols.

### 2.5. Ethics Approval

The study was conducted with a strong emphasis on safeguarding the safety, dignity, and well-being of all participants, recognizing that individuals with sarcopenia represent a particularly vulnerable population. Participants were thoroughly informed about the study’s objectives, potential risks and benefits, and the strict measures implemented to ensure data confidentiality, in full compliance with data protection regulations. They were also made aware of their right to withdraw from the study at any point, without the need to provide justification and without any negative consequences.

Written informed consent was obtained from all participants following comprehensive, clear, and age-appropriate explanations, with researchers addressing all patient questions in detail. The study adhered strictly to the ethical principles outlined in the Declaration of Helsinki and followed the Good Clinical Practice (GCP) guidelines. Ethical approval for the study was granted by the Ethics Committee of the University of Medicine and Pharmacy of Craiova, under approval number 204/20 September 2023.

### 2.6. Statistical Analysis

To ensure methodological consistency, all statistical procedures and related parameters referenced throughout the methodology are detailed below, including reporting format, sample size calculation, and effect size estimation.

Statistical analysis was performed using Microsoft Excel 365 (2021, free software, Microsoft Corporation, Washington, DC, USA), SPSS Statistics version 26.0 (IBM Corp., Armonk, NY, USA), and the XLSTAT add-on for Microsoft Excel (Addinsoft SARL, Paris, France). Data were initially recorded in Excel and subsequently imported into SPSS and XLSTAT for advanced analysis.

The distribution of variables was tested using the Anderson–Darling normality test, which revealed a non-Gaussian distribution. Accordingly, non-parametric tests were used throughout. All analyses were two-tailed, and a *p*-value < 0.05 was considered statistically significant.

Quantitative variables were expressed as mean ± standard deviation (SD), or median with interquartile range (IQR: 25th–75th percentiles), depending on distribution. Qualitative variables were expressed as absolute and relative frequencies.

For comparison:

- Within-group differences (T1 vs. T2) were analyzed using the Wilcoxon signed-rank test;
- Between-group differences (SG vs. CG) were assessed with the Mann–Whitney U test;
- Associations between continuous variables were explored using Spearman’s rank correlation coefficient (ρ).

To enhance the interpretation of results, effect sizes were calculated using Cohen’s d, with thresholds interpreted as follows: small (d = 0.2), medium (d = 0.5), and large (d ≥ 0.8).

Additionally, a sample size calculation was performed based on the Wilcoxon signed-rank test using the formula:

N = (Z α/2+Z β)/d

where

- Zα/2 = 1.96 (for a two-tailed test at α = 0.05);
- Zβ = 0.84 (for 80% power);
- d = 0.843 (expected effect size based on prior studies using SPPB and SarQoL.

This yielded a required minimum sample of 12 participants per group. With 40 participants in each group, the final sample was sufficiently powered to detect meaningful differences.

During data analysis it also used Julius AI-an assistant platform powered by the GPT-4 model from OpenAI. For data processing and graphics, within this platform it was used Python version 3.8.

## 3. Results

### 3.1. Baseline Patient Characteristics

The baseline characteristics of the study participants, providing an overview of their demographic, anthropometric, and clinical data, are summarized in Table 3. A detailed analysis of each category is provided below to highlight any initial differences between the study and control groups.

#### 3.1.1. Demographic and Anthropometric Characteristics

The average age was comparable between the two groups: 72.45 ± 4.24 years in the Study Group (SG) and 72.32 ± 4.48 years in the Control Group (CG), with no statistically significant difference (*p* = 0.898). All participants were aged between 68 and 81 years.

The urban-to-rural ratio was 20:20 in the SG and 18:22 in the CG, showing no significant differences (*p* = 0.822 for urban participants and *p* = 0.659 for rural participants). Additionally, no statistically significant differences were found in BMI classification between the groups (*p* = 0.328), confirming the homogeneity of the study population.

**Figure 2:**
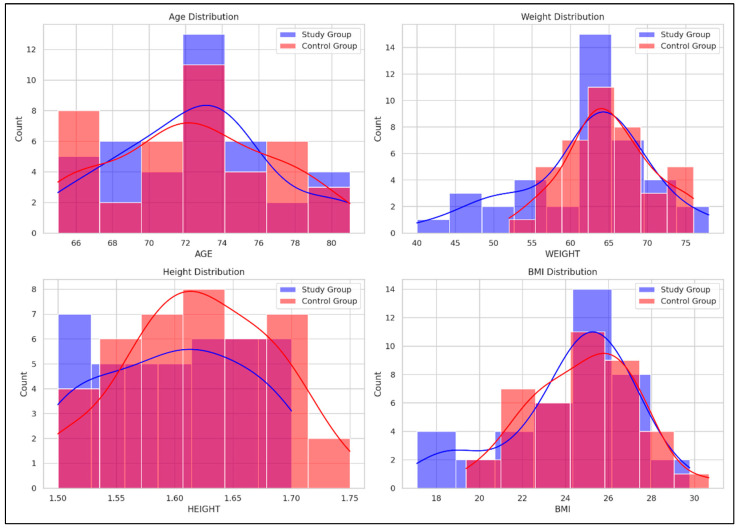
Comparative distribution of age, weight, height, and BMI between the Study and Control groups. Red curve = the density distribution for the SG, Blue curve = the density distribution for the CG.

Figure 2 illustrates comparative histograms with overlaid density curves for age, weight, height, and BMI between the SG and CG. The SG demonstrated more concentrated distributions across all variables, indicating less variability within this group, while the CG showed broader distributions, indicating greater variability within this group.

- Age: SG peaked around 70–75 years, while CG showed a broader range (66–80 years);
- Weight: SG peaked at 60–65 kg; CG showed a broader distribution with a peak at 65–70 kg;
- Height: SG peaked around 1.65–1.70 m; CG had higher counts in the 1.60–1.65 m range;
- BMI: SG showed a peak at 24–26 kg/m^2^, while CG had a broader distribution with peaks around 26–28 kg/m^2^.

To complement the numerical data presented in Table 3, Figure 2 offers a visual representation of the baseline characteristics (age, weight, height, and BMI) for both SG and CG. This graphical overview supports a more intuitive understanding of the comparability between groups at the beginning of the intervention and allows for a rapid visual assessment of potential distributional differences that may not be immediately evident from tabulated values alone.

#### 3.1.2. Functional Outcomes and Quality of Life Assessment

The SPPB scores were comparable between groups (5.75 ± 0.86 in SG vs. 5.8 ± 0.88 in CG; *p* = 0.798), indicating similar physical performance levels at baseline.

The SarQoL scores revealed lower quality of life in both groups. The SG had an average score of 54.42 ± 8.76, while the CG scored 55.59 ± 4.61, with no significant difference between groups (*p* = 0.457).

These results suggest that participants in both groups experience a similarly reduced quality of life, consistent with the expected impact of sarcopenia.

### 3.2. Study Group, Time-Evolution

**Figure 3:**
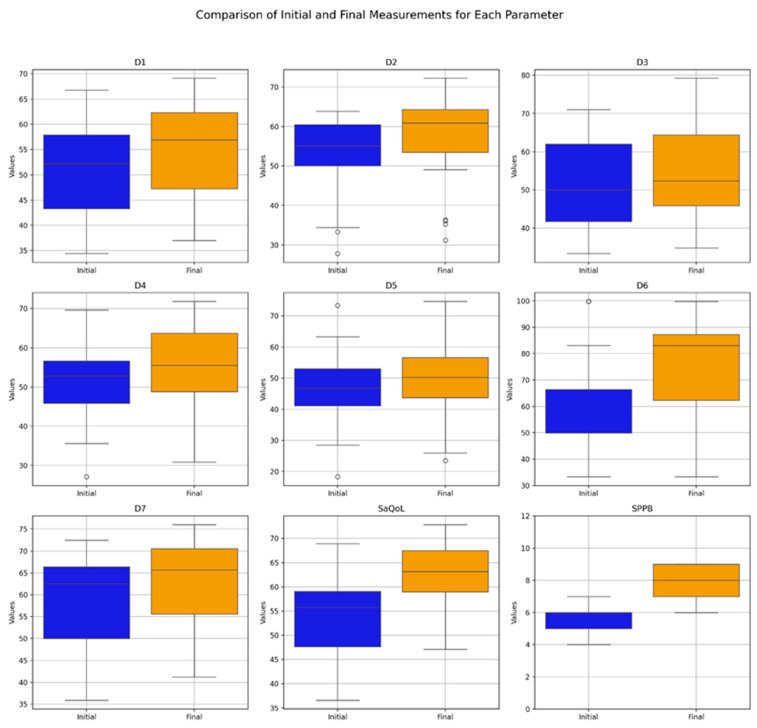
Comparative analysis of initial vs. final parameters in the Study Group (Boxplot Visualization), *p*-values derived from the Wilcoxon signed-rank test. D1 = Physical and mental health, D2 = Locomotion, D3 = Body composition, D4 = Functionality, D5 = Activities of daily living, D6 = Leisure activities, D7 = Fears, SaQoL = SaQoL questionnaire, SPPB = short physical performance battery.

Table 4 presents the values of the analyzed parameters, focusing on their progression between the initial (T1) and final (T2) assessments. The parameters assessed include the sarcopenia quality of life (SarQoL) questionnaire—covering total scores and domain-specific scores (D1–D7)—and the short physical performance battery (SPPB). The Wilcoxon signed-rank test was employed to evaluate changes over time, revealing statistically significant improvements across all measured parameters (Figure 3).

The boxplot in Figure 3 provides a visual summary of the within-group changes, illustrating the distribution and variability of key outcome measures before and after the intervention in the Study Group. The Wilcoxon signed-rank test results indicate significant changes in all domains of the SaQoL questionnaire (D1–D7) and the SPPB, with all *p*-values falling below the 0.05 significance threshold.

Specifically:

- Strong Improvements: Physical and mental health (D1), locomotion (D2), functionality (D4), fears (D7), SaQoL total score, and SPPB showed highly significant improvements (*p* < 0.001), suggesting strong evidence against the null hypothesis and reflecting the substantial positive impact of the intervention;
- Moderate Improvements: Body composition (D3) and activities of daily living (D5) also exhibited significant improvements, though to a slightly lesser extent compared to the parameters above;
- Notable Changes in Leisure Activities (D6): While D6 showed significant improvement, the *p*-value suggests moderate evidence against the null hypothesis, indicating variability in how leisure activities responded to the intervention.

**Figure 4:**
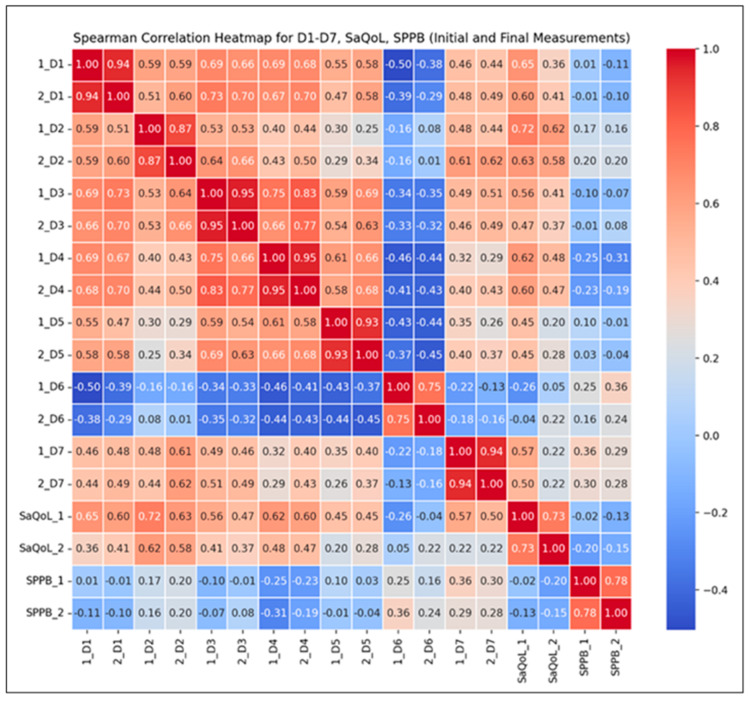
Spearman correlation heatmap for the Study Group.

To further validate the intervention’s effectiveness, Spearman correlation analyses (Figure 4) were conducted to examine the relationships between the total SarQoL score, its individual domains, and the SPPB results. These analyses aim to highlight how improvements in physical performance are associated with specific dimensions of health-related quality of life.

Correlation Findings:

- Strong Positive Correlations (r ≥ 0.8): Observed across most SarQoL domains (D1–D7), indicating that higher initial scores generally predict higher final scores, suggesting consistent improvements over time in sarcopenia-related quality of life;
- Moderate Positive Correlations (r = 0.5–0.8): Found between total SarQoL and SPPB scores, reflecting a positive but more variable trend in functional performance and quality of life improvements;
- Notable Inter-domain Correlations: For instance, the correlation between initial locomotion scores (D2) and final SarQoL outcomes (r = 0.58) suggests that early physical performance may moderately predict quality of life improvements post-intervention.

### 3.3. Control Group, Time-Evolution

Table 5 presents the values of the studied parameters for the Control Group (CG). The results from the Wilcoxon signed-rank test indicate statistically significant differences between the initial (T1) and final (T2) measurements for D3 (body composition), D5 (activities of daily living), and D7 (fears) from the SarQoL questionnaire, while the remaining parameters did not show significant changes. 

A notable observation is the consistently low score in D7 (fears), which may explain the lack of adherence to rehabilitation programs in this group, as fear-related factors could influence motivation and engagement.

**Figure 5:**
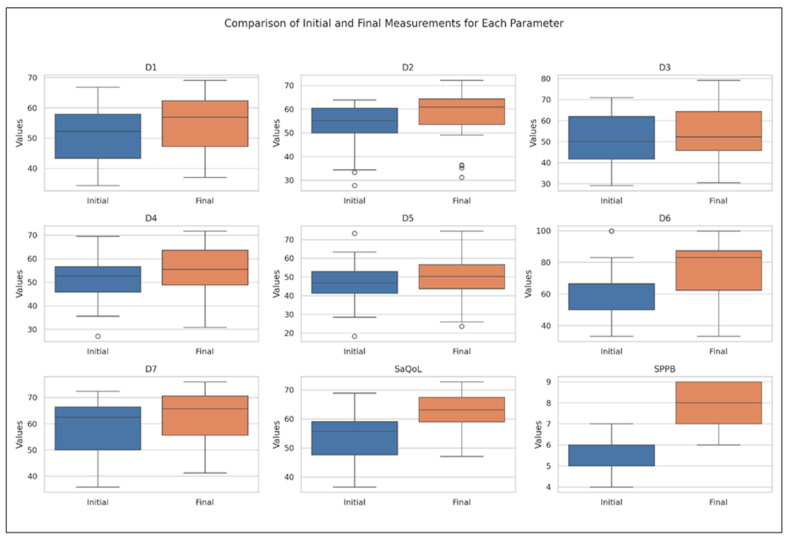
Comparative analysis of initial vs. final parameters in Control Group: insights from Boxplot Visualization. The Wilcoxon signed rank test was used for *p*-values, D1 = Physical and mental health, D2 = Locomotion, D3 = Body composition, D4 = Functionality, D5 = Activities of daily living, D6 = Leisure activities, D7 = Fears, SaQoL = SaQoL questionnaire, SPPB = short physical performance battery.

Figure 5 offers a graphical overview of the changes observed within the CG, highlighting the distribution patterns and variability of key outcome indicators measured before and after the intervention. While most parameters show minimal changes, D3 (body composition), D5 (activities of daily living), and D7 (fears) exhibit visible improvements, which were statistically significant.

**Figure 6:**
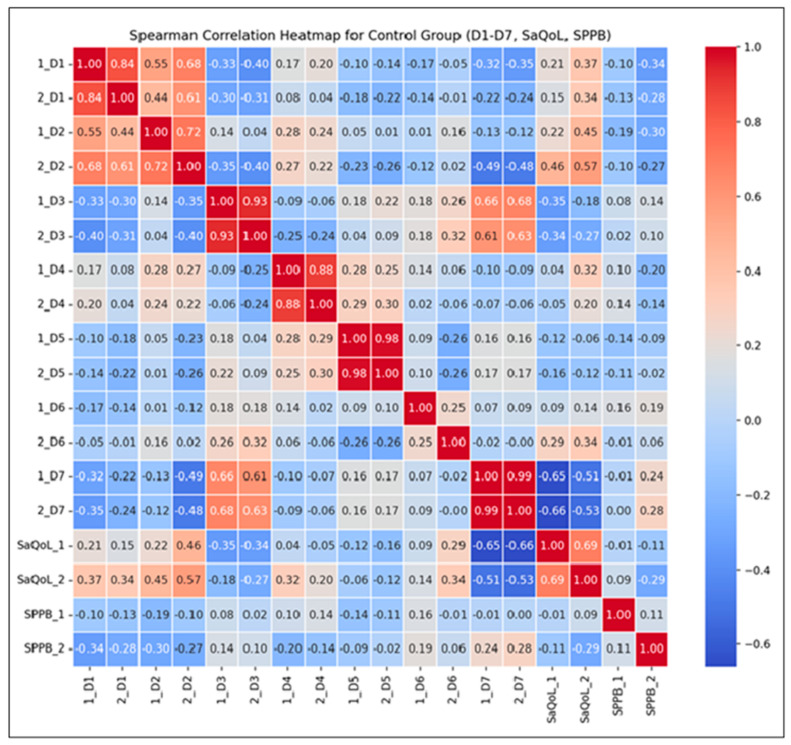
Spearman correlation heatmap for the Control Group.

The Spearman correlation heatmap (Figure 6) illustrates the strength and direction of monotonic relationships between the initial (T1) and final (T2) measurements.

Key Observations:

- Strong Positive Correlations: High correlations (r > 0.7) between D1–D7 suggest consistent scores over time, reflecting the stability of these parameters without intervention. This consistency implies that the natural course of the condition, without therapeutic input, leads to minimal fluctuations;
- Moderate Correlations for the SarQoL Total Score (r = 0.69): This indicates some variability in quality of life, likely influenced by daily life factors rather than structured intervention;
- Low Correlation for SPPB (r = 0.11): This suggests no predictable change in physical performance over time, as no supervised kinetic measures were performed. This highlights the importance of active rehabilitation in maintaining or improving physical function.

The correlations between the initial and final measurements of D1 through D7 are generally strong, indicating that the scores are consistent over time within the CG. For SaQoL scores, the correlation between initial and final (r = 0.69) is moderate, suggesting some variability but generally indicating that quality of life remains consistent. This moderate correlation shows that while there may be some changes in quality of life, they are not drastic, due to the absence of a rehabilitation program. The correlation between initial and final SPPB scores (r = 0.11) is very low, indicating that there is little to no predictable change in physical performance; no patient of CG failed to perform any supervised kinetic measures. This suggests that the SPPB scores vary more independently of their initial values compared to other parameters.

### 3.4. Study Group Versus Control Group

The comparison between the Study Group (SG) and the Control Group (CG) was conducted using the Mann–Whitney U test, which evaluates differences in the distribution of parameters between the two groups. The U statistics and *p*-values for each variable provide insight into the impact of the rehabilitation program on sarcopenia-related outcomes.

Initial Comparisons (T1):

- Parameters with significant differences (*p* < 0.05): D1 (physical and mental health) (*p* = 0.046), D2 (locomotion) (*p* = 9.72 × 10^−8^), D4 (functionality) (*p* = 0.044), D7 (fears) (*p* = 5.42 × 10^−7^). These results suggest that the SG experienced notable improvements in these domains, likely due to the initial impact of the intervention;
- Parameters with non-significant differences (*p* ≥ 0.05): The SaQoL total score (*p* = 0.095) and SPPB (*p* = 0.787) showed no significant differences, indicating similar baseline distributions. D3 (body composition) (*p* ≈ 0.358), D5 (activities of daily living) (*p* = 0.488), and D6 (leisure activities) (*p* = 0.095) also showed no significant differences, reflecting baseline homogeneity.

**Figure 7:**
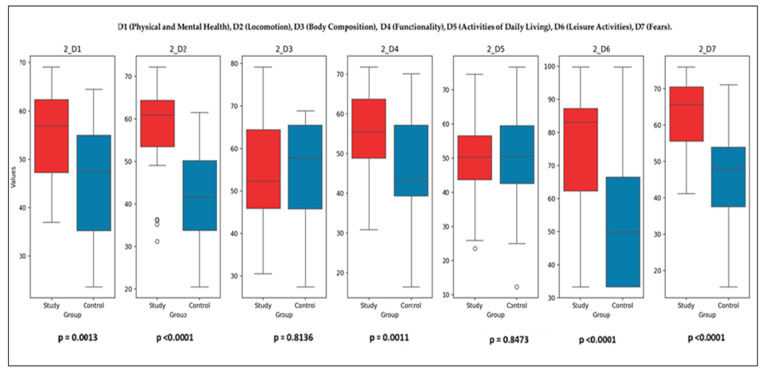
Final comparison of SaQoL domains (D1–D7) and SPPB between Study and Control groups.

Post-Intervention Comparisons (T2): Figure 7 illustrates the final comparison between the SG and CG across all SaQoL domains (D1–D7) and SPPB. Bulleted lists look like the following:

- Parameters with Significant Differences (*p* < 0.05): D1 (physical and mental health), D2 (locomotion), D4 (functionality), D6 (leisure activities), D7 (fears), and SaQoL total score (*p* = 9.78 × 10^−6^) showed highly significant improvements in the SG compared to the CG. SPPB (short physical performance battery) demonstrated an extremely significant difference (*p* = 2.01 × 10^−11^), highlighting the profound effect of the rehabilitation program on physical performance;
- Parameters with No Significant Differences (*p* ≥ 0.05): D3 (body composition) (*p* > 0.05) and D5 (activities of daily living) (*p* > 0.05) did not show significant changes, suggesting that these parameters were less influenced by the intervention.

While the SG demonstrated statistically significant improvements in both SPPB and SarQoL scores between baseline and follow-up, not all domain-level comparisons reached statistical significance when evaluated between groups (SG vs. CG). This may be due to the relatively small sample size and intra-group variability, particularly in certain SarQoL subdomains such as D3 and D5. Additionally, slight improvements observed in CG—likely due to natural recovery or behavioral adjustments—may have narrowed the between-group contrast. These findings highlight the sensitivity of within-group analyses in detecting individual response to intervention, while also underscoring the importance of effect size and clinical relevance, not solely *p*-values, when interpreting group differences.

## 4. Discussion

This study represents a pioneering effort to evaluate the impact of a structured rehabilitation program on the QoL in patients over 65 years of age with primary sarcopenia. Unlike most previous research, which primarily utilized cross-sectional designs to assess HRQoL at a single time point [53], our controlled prospective study incorporated longitudinal elements, monitoring changes over a six-month period. This approach provided valuable insights into the dynamic effects of rehabilitation on both clinical and functional outcomes.

The baseline characteristics of the participants revealed no significant differences between SG and CG, confirming the homogeneity of the study population concerning key variables such as age, BMI, and initial SarQoL scores. This homogeneity strengthens the internal validity of our findings, minimizing potential confounding factors. Both groups exhibited similarly reduced baseline SarQoL scores, reflecting the adverse impact of sarcopenia on daily living, consistent with findings from studies conducted in Turkey and Spain [54,55,56]. However, the initial domain-specific scores were lower in our cohort, possibly due to lifestyle factors unique to our population.

Our rehabilitation strategy, combining kinesiotherapy and deep oscillation therapy, was instrumental in driving positive outcomes. The six-month program, aligned with ESCEO guidelines [57], included resistance and aerobic training tailored to the specific needs of older adults with sarcopenia. The significant improvements observed in physical and mental health, locomotion, functionality, and reduction in fears are particularly noteworthy. These results corroborate meta-analyses conducted by Lu et al. (2021) and Shen et al. (2023), which highlighted the efficacy of combined kinesiotherapy in enhancing physical performance in older adults with sarcopenia [31,32].

Deep oscillation therapy, applied for the first time in this context, contributed to gains in muscle function and QoL. While previous studies on vibration therapy have shown mixed results [58], our findings support its role as an effective adjunct to conventional rehabilitation. This discrepancy may be attributed to differences in therapy protocols, such as frequency, intensity, and the integration with resistance training, as suggested by Wu et al. (2020) [58]. The improvements in leisure activities (D6) and reduction in fears (D7) further underscore the holistic benefits of our intervention, enhancing both physical capabilities and psychological well-being. Similar approaches have been documented in other musculoskeletal disorders, where rehabilitation modalities, including kinesiotherapy, play a crucial role in pain management and functional improvement [58,59,60,61].

Following the rehabilitation program, SG demonstrated statistically significant improvements across all SarQoL domains and total scores. Notably, physical and mental health (D1), locomotion (D2), functionality (D4), and activities of daily living (D5) showed marked enhancements, consistent with previous studies highlighting the benefits of exercise-based interventions [31,32]. The total SarQoL score increased significantly, surpassing the moderate risk threshold for sarcopenia-related QoL impairment [62]. In contrast, CG exhibited minimal changes, with significant improvements observed only in body composition (D3), activities of daily living (D5), and fears (D7), likely attributable to natural lifestyle variations rather than the effects of a structured intervention. These findings are in line with previous research on the rehabilitative outcomes or recovery of physical performance of elderly patients, where a multidisciplinary approach, including rehabilitation programs, has been shown to improve patient outcomes in conditions such as osteoporosis-related fractures [63]. The need for comprehensive therapeutic strategies is further supported by evidence from studies on chronic musculoskeletal conditions, highlighting the benefits of targeted rehabilitation techniques [60,61,63].

Despite the overall positive outcomes, certain domains, particularly body composition and activities of daily living, exhibited less pronounced improvements. These findings align with meta-analyses conducted by Rondanelli et al. (2020), which suggested that achieving significant changes in body composition often requires longer intervention periods coupled with nutritional support [34]. Similarly, Giallauria et al. (2016) emphasized that while resistance training effectively enhances muscle strength and physical function, its impact on muscle mass may be limited without the inclusion of dietary interventions [35]. Furthermore, the modest improvements observed in D5 may reflect the inherent complexity of daily living activities, which are influenced not only by physical capacity but also by cognitive, emotional, and environmental factors. This observation is supported by Wu et al. (2020), who reported that physical interventions alone might not be sufficient to produce substantial changes in daily functioning unless accompanied by concurrent psychosocial support [58]. This underscores the importance of adopting a multidimensional rehabilitation approach that integrates physical training with psychological and social interventions to optimize outcomes in activities of daily living.

The significant improvements observed in the SPPB scores in SG indicate a shift from moderate to minor physical performance limitations. This improvement aligns with the findings of Wu et al. (2020) and Zhao et al. (2022), who demonstrated the positive effects of resistance and vibration training on physical function in older adults [58,64]. In contrast, CG showed negligible improvement, highlighting the critical role of structured rehabilitation programs in achieving meaningful improvements in physical performance and HRQoL.

The observed correlations between SPPB and the SarQoL domains highlight the interdependence between physical performance (as measured by SPPB) and QoL in sarcopenic patients. Although these associations may be expected, their statistical confirmation supports the internal consistency of the dataset and aligns with the study’s objective to evaluate multidimensional outcomes. These findings emphasize that improvements in physical function are closely tied to perceived well-being, reinforcing the value of comprehensive, patient-centered rehabilitation strategies. Additionally, our SPPB results support the cut-off values proposed by Cesari et al. (2015) [65] and the AWGS [6], confirming the tool’s sensitivity in detecting post-intervention changes in physical performance. This validation underscores the utility of SPPB as a reliable assessment tool for monitoring functional outcomes in sarcopenic populations.

Post-intervention comparisons between the SG and CG revealed significant differences in key SarQoL domains (D1, D2, D4, D6, D7) and the total SarQoL score, reinforcing the effectiveness of the rehabilitation program. Interestingly, body composition (D3) and activities of daily living (D5) showed less responsiveness, suggesting that longer interventions or supplementary therapies (e.g., nutritional supplementation or cognitive-behavioral therapy) may be required to target these areas effectively.

Our findings are consistent with meta-analyses by Rondanelli et al. (2020) and Giallauria et al. (2016), which highlighted the role of resistance training in improving muscle strength and physical performance [34,35]. However, the limited impact on body composition aligns with Wu et al.’s (2020) observation that vibration therapy may not significantly affect muscle mass despite functional gains [58].

Study Limitations and Generalizability: Several limitations of this study should be considered when interpreting the findings. First, the study sample consisted exclusively of older women, which limits the generalizability of the results to other populations, such as older men or individuals with secondary sarcopenia. Second, the non-randomized design may have introduced selection bias; however, we attempted to mitigate this through strict inclusion/exclusion criteria, baseline comparability, and statistical adjustments. Third, while the study was adequately powered, the relatively small sample size may have reduced the sensitivity to detect certain between-group differences, especially in subdomains such as body composition or activities of daily living. Additionally, adherence to the intervention—although high overall—may have varied among participants, potentially affecting outcome consistency. Finally, the single-center design and controlled conditions may limit the external validity of the results, as real-world rehabilitation settings may differ in terms of resources, supervision, and patient engagement. Future research in more diverse, multicenter cohorts is warranted to validate and extend these findings.

## 5. Conclusions

This study underscores the multidimensional benefits of a structured rehabilitation program for older primary sarcopenia patients. The integration of kinesiotherapy and deep oscillation therapy led to significant improvements in QoL and physical performance, with positive effects observed across most SarQoL domains and SPPB scores. These findings highlight the importance of comprehensive, patient-centered rehabilitation strategies in managing sarcopenia and improving the overall well-being of affected individuals.

Future research should explore the long-term sustainability of these benefits and investigate complementary interventions to address less responsive domains, such as body composition and daily living activities. Additionally, larger multicenter trials could validate our results and contribute to the development of standardized rehabilitation protocols for sarcopenic populations.

## Figures and Tables

**Table 1:** In-hospital and outpatient rehabilitation program (Stages 1 and 3).

	Components	Description
Stage 1—In-Hospital Kinesiotherapy and Deep Oscillation Therapy	Kinesiotherapy - resistance exercises, daily, 15 min - balance and gait training, daily, 15 min - low intensity aerobic training (endurance training) performed at 40–60% of maximum heart rate, according to the equation ((220 − age) × 0.65), three times a week, 40 min	Resistance exercises (strength training) We selected agonist-antagonist muscle groups—knee and elbow flexors-extensors). Both sides with a possible full range of motion. Exercise at a load around 40–50% of baseline strength as the initial training stimulus. A total of 8–12 repetitions, 3 sets, separated by rest intervals of 90 s. We increased resistance by adding small weights or using elastic bands (TheraBand Latex Resistance Bands), and body resistance. Balance and gait training—mono and bipedal walking on an unstable proprioceptive platform. Resistance and balance and gait training were performed a.m. Endurance training (aerobic training)—many repetitions, low resistance. We selected an aerobic exercise (walking, cycling) using large muscle groups. Begin with low-intensity exercise (40% of maximum heart rate) for a short duration (5–10 min). Then, the intensity of exercise was maintained at 60% of maximum heart rate. We included warm-up and cool-down exercises (stretching and breathing exercises). The aerobic program was performed p.m.
	Deep oscillation—therapy with manual applicator 30 min, daily	First, 5 min—high frequency 100 Hz, for the thixotropic, analgesic, and muscle-relaxing effect. Then, 10 min—low frequency 5–25 Hz, which activates local metabolism, improving muscle function. A 5 cm oscillator head was applied to both thigh muscles.
The second stage (5 months)—Table 2 Home training kinetic program, with weekly monitoring through the outpatient service		
Stage 3—Outpatient Kinesiotherapy and Deep Oscillation Therapy	Kinesiotherapy - functional and balance training - aerobic endurance training 30–40 min	Implement balance training such as standing on one foot, walking heel-to-toe, or using balance boards to reduce fall risk and improve body coordination. (10 min) Practice functional movements that mimic daily activities, such as stepping up and down stairs, sitting down and standing up from a chair, and carrying weights to simulate grocery bags (10 min). Pedaling on the elliptical bike. Begin with short durations (10–15 min) and gradually increase to 20 min.
	Deep oscillation—therapy with manual applicator 30 min, daily	First, 5 min—high frequency 100 Hz, for the thixotropic, analgesic, and muscle-relaxing effect. Then, 10 min—low frequency 5–25 Hz, which activates local metabolism, improving muscle function. A 5 cm oscillator head was applied to both thigh muscles.

**Table 2:** Weekly home-training program in SG patients (Stage 2).

Day	Type of Training	Activities	Duration
Day 1	Aerobic Training	Warm-up (10 min): gentle stretching and slow walking or stationary cycling. Main activity (20 min): walking on a flat surface or treadmill. Cool-down (10 min): slow walking and stretching.	40 min
Day 2	Resistance training	Warm-up (10 min): light stretching and mobility exercises. Main activity (20 min): seated leg press, arm curls with resistance bands, chest press. Cool-down (10 min): gentle stretching.	40 min
Day 3	Balance training	Warm-up (10 min): gentle stretching of the upper and lower body. Main activity (20 min): Single-leg stands: hold each leg for up to 30 s, using a chair for support if needed. Heel-to-toe walk: walk in a straight line, placing the heel of one foot directly in front of the toes of the other foot. Standing yoga poses: tree pose or warrior III, using a wall or chair for support. Cool-down (10 min): gentle stretching focusing on the legs and lower back.	40 min
Day 4	Rest day	Activity: Light walking or leisure activities (gardening, shopping, light housework)	40–60 min
Day 5	Combined aerobic and light resistance training	Warm-up (10 min): gentle stretching and mobility exercises. Main activity (40 min): cycling on a stationary bike, light resistance circuit. Cool-down (10 min): stretching and relaxation exercises.	60 min
Day 6	Flexibility training	Warm-up (10 min): light cardiovascular exercise like walking or stationary cycling at a very low intensity. Main Activity (20 min): Dynamic stretches: leg swings and arm circles to improve range of motion. Static stretches: hold stretches for each major muscle group for 20–30 s, such as hamstring and quadriceps stretches, and arm stretches. Cool-down (10 min): deep breathing and relaxation techniques to enhance muscle relaxation.	40 min
Day 7	Rest day	Activity: light, non-strenuous activities such as walking around the home or gardening, gentle walking in a park with low-intensity movements	30–60 min

**Table 3:** Baseline characteristics assessment.

	Study Group 40 Females	Control Group 40 Females	*p*-Value
Age (years)	72.45 ± 4.24	72.32 ± 4.48	0.898
Weight (kg)	61.97 ± 8.42	65.07 ± 5.79	0.059
Height (m)	1.59 ± 0.06	1.62 ± 0.06	0.125
BMI (kg/m^2^)	24.23 ± 2.94	24.82 ± 2.41	0.328
Urban (*n*, %)	20 (50%)	18 (45%)	0.822
Rural (*n*, %)	20 (50%)	22 (55%)	0.659
SPPB	5.75 ± 0.86	5.8 ± 0.88	0.798
SarQoL	54.42 ± 8.76	55.59 ± 4.61	0.457

Variables are reported as mean ± (SD) (standard deviation), *n* = number of subjects, % = percent of patients. BMI = Body Mass IndexSarQoL = SarQoL questionnaire, SPPB = short physical performance battery, *p* = *t*-test for independent samples.

**Table 4:** Study group parameters at initial and final assessments.

Parameters		Mean Value	SD	Min Value	25th Percentile	Median Value	75th Percentile	Max Value	*p* Value
D1 = Physical and mental health	T1	51.02	9.18	34.40	43.30	52.20	57.92	66.80	<0.001
	T2	54.86	9.50	37.00	47.25	56.90	62.35	69.10	
D2 = Locomotion	T1	53.50	9.00	27.80	50.00	55.10	60.50	63.90	<0.001
	T2	57.86	9.71	31.20	53.47	60.90	64.35	72.20	
D3 = Body composition	T1	50.20	11.17	29.20	41.70	50.00	61.97	71.00	<0.001
	T2	54.50	12.31	30.50	45.87	52.30	64.40	79.20	
D4 = Functionality	T1	51.62	9.33	27.10	45.87	52.80	56.65	69.60	<0.001
	T2	55.90	9.78	30.80	48.82	55.50	63.70	71.80	
D5 = Activities of daily living	T1	47.13	10.11	18.30	41.15	46.80	53.00	73.30	<0.001
	T2	50.63	10.82	23.50	43.72	50.30	56.60	74.60	
D6 = Leisure activities	T1	59.03	19.88	33.30	49.90	49.90	66.50	99.80	<0.001
	T2	76.07	19.52	33.30	62.35	83.10	87.27	99.80	
D7 = Fears	T1	59.60	8.57	35.90	50.00	62.50	66.40	72.40	<0.001
	T2	63.45	8.38	41.20	55.57	65.65	70.55	76.00	
Total SaQoL	T1	54.42	8.76	36.60	47.62	55.75	59.07	68.90	<0.001
	T2	62.55	7.00	47.10	58.95	63.15	67.50	72.80	
SPPB	T1	5.75	0.86	4.00	5.00	6.00	6.00	7.00	<0.001
	T2	8.05	0.90	6.00	7.00	8.00	9.00	9.00	

T1 = Initial, T2 = Final, D (D1–D7) = domains of SaQoL questionnaire, SaQoL = SaQoL questionnaire, SPPB = short physical performance battery, *p* = *t*-test for independent samples.

**Table 5:** Control Group parameters at initial and final assessments.

Parameters		Mean Value	SD	Min Value	25th Percentile	Median Value	75th Percentile	Max Value	*p* Value
D1 = Physical and mental health	T1	55.48	5.39	42.30	51.65	55.15	58.35	68.30	0.5537
	T2	54.14	5.42	43.40	50.57	54.45	57.22	64.50	
D2 = Locomotion	T1	44.14	6.63	30.20	41.52	44.90	46.70	58.90	0.6957
	T2	45.56	7.91	31.30	40.47	44.30	50.22	61.50	
D3 = Body composition	T1	54.45	9.02	38.60	47.47	55.60	63.42	67.10	<0.001
	T2	56.47	9.02	42.00	46.40	57.80	65.50	68.90	
D4 = Functionality	T1	50.40	9.55	35.20	42.40	48.30	57.70	69.70	0.1498
	T2	50.38	9.63	29.80	43.00	49.20	57.12	69.10	
D5 = Activities of daily living	T1	51.37	9.38	40.70	43.77	48.20	57.82	74.20	<0.001
	T2	52.80	10.08	35.00	45.15	50.65	59.55	76.70	
D6 = Leisure activities	T1	52.81	21.25	33.30	33.30	49.90	66.50	99.80	0.4805
	T2	56.13	20.49	33.30	33.30	49.90	66.50	99.80	
D7 = Fears	T1	44.06	14.55	12.80	36.77	46.20	52.62	70.60	<0.001
	T2	45.52	14.17	15.50	37.55	47.80	53.97	71.10	
Total SaQoL	T1	55.59	4.61	48.60	51.77	56.65	58.82	68.10	0.1528
	T2	56.51	5.51	43.60	52.45	56.35	58.97	69.20	
SPPB	T1	5.80	0.88	4.00	5.00	6.00	6.00	7.00	0.4113
	T2	6.17	0.78	5.00	6.00	6.00	7.00	8.00	

T1 = Initial, T2 = Final, D (D1–D7) = domains of SaQoL questionnaire, SaQoL = SaQoL questionnaire, SPPB = short physical performance battery, *p* = *t*-test for independent samples.

## Data Availability

Data are contained within the article.

## References

[1] Piastra G., Perasso L., Lucarini S., Monacelli F., Bisio A., Ferrando V., Gallamini M., Faelli E., Ruggeri P. (2018). Effects of Two Types of 9-Month Adapted Physical Activity Program on Muscle Mass, Muscle Strength, and Balance in Moderate Sarcopenic Older Women. BioMed Res. Int..

[2] Wang H., Huang W.Y., Zhao Y. (2022). Efficacy of Exercise on Muscle Function and Physical Performance in Older Adults with Sarcopenia: An Updated Systematic Review and Meta-Analysis. Int. J. Environ. Res. Public Health.

[3] Dufour A.B., Hannan M.T., Murabito J.M., Kiel D.P., McLean R.R. (2013). Sarcopenia Definitions Considering Body Size and Fat Mass Are Associated with Mobility Limitations: The Framingham Study. J. Gerontol. A Biol. Sci. Med. Sci..

[4] Pan L., Xie W., Fu X., Lu W., Jin H., Lai J., Zhang A., Yu Y., Li Y., Xiao W. (2021). Inflammation and Sarcopenia: A Focus on Circulating Inflammatory Cytokines. Exp. Gerontol..

[5] Damanti S., Senini E., De Lorenzo R., Merolla A., Santoro S., Festorazzi C., Messina M., Vitali G., Sciorati C., Rovere-Querini P. (2024). Acute Sarcopenia: Mechanisms and Management. Nutrients.

[6] Chen L.K., Woo J., Assantachai P., Auyeung T.W., Chou M.Y., Iijima K., Jang H.C., Kang L., Kim M., Kim S. (2020). Asian Working Group for Sarcopenia: 2019 Consensus Update on Sarcopenia Diagnosis and Treatment. J. Am. Med. Dir. Assoc..

[7] Sutil D.V., Parentoni A.N., Da Costa Teixeira L.A., de Moreira B.S., Leopoldino A.A.O., Mendonça V.A., Lacerda A.C.R., Danielewicz A.L., de Avelar N.C.P. (2023). Prevalence of Sarcopenia in Older Women and Level of Agreement between the Diagnostic Instruments Proposed by the European Working Group on Sarcopenia in Older People 2 (EWGSOP2). BMC Musculoskelet. Disord..

[8] Yadigar S., Yavuzer H., Yavuzer S., Cengiz M., Yürüyen M., Döventaş A., Erdinçler D.S. (2016). Primary Sarcopenia in Older People with Normal Nutrition. J. Nutr. Health Aging.

[9] Kawada T. (2021). Mortality Risk of Sarcopenia in Older Subjects. J. Am. Med. Dir. Assoc..

[10] Xu J., Wan C.S., Ktoris K., Reijnierse E.M., Maier A.B. (2021). Sarcopenia Is Associated with Mortality in Adults: A Systematic Review and Meta-Analysis. Gerontology.

[11] Lee G.K.Y., Au P.C.M., Li G.H.Y., Chan M., Li H.L., Cheung B.Y.M., Wong I.C.K., Lee V.H.F., Mok J., Yip B.H.K. (2021). Sarcopenia and Mortality in Different Clinical Conditions: A Meta-Analysis. Osteoporos. Sarcopenia.

[12] Cruz-Jentoft A., Landi F., Schneider S., Zúñiga C., Arai H., Boirie Y., Chen L.K., Fielding R.A., Martin F.C., Michel J.P. (2014). Prevalence of and Interventions for Sarcopenia in Ageing Adults: A Systematic Review. Age Ageing.

[13] Cristea A., Qaisar R., Edlund P.K., Lindblad J., Bengtsson E., Larsson L. (2010). Effects of Aging and Gender on the Spatial Organization of Nuclei in Single Human Skeletal Muscle Cells. Aging Cell.

[14] Combaret L., Dardevet D., Béchet D., Taillandier D., Mosoni L., Attaix D. (2009). Skeletal Muscle Proteolysis in Aging. Curr. Opin. Clin. Nutr. Metab. Care.

[15] Sandri M. (2010). Autophagy in Skeletal Muscle. FEBS Lett..

[16] Brook M.S., Wilkinson D.J., Phillips B.E., Perez-Schindler J., Philp A., Smith K., Atherton P.J. (2016). Skeletal Muscle Homeostasis and Plasticity in Youth and Ageing: Impact of Nutrition and Exercise. Acta Physiol..

[17] Jiao J., Demontis F. (2017). Skeletal Muscle Autophagy and Its Role in Sarcopenia and Organismal Aging. Curr. Opin. Pharmacol..

[18] Léger B., Derave W., De Bock K., Hespel P., Russell A.P. (2008). Human Sarcopenia Reveals an Increase in SOCS-3 and Myostatin and a Reduced Efficiency of Akt Phosphorylation. Rejuvenation Res..

[19] Franceschi C., Garagnani P., Parini P., Giuliani C., Santoro A. (2018). Inflammaging: A New Immune-Metabolic Viewpoint for Age-Related Diseases. Nat. Rev. Endocrinol..

[20] Short K.R., Bigelow M.L., Kahl J., Singh R., Coenen-Schimke J., Raghavakaimal S., Nair K.S. (2005). Decline in Skeletal Muscle Mitochondrial Function with Aging in Humans. Proc. Natl. Acad. Sci. USA.

[21] Kemp P.R., Griffiths M., Polkey M.I. (2019). Muscle Wasting in the Presence of Disease, Why Is It so Variable?. Biol. Rev. Camb. Philos. Soc..

[22] Beaudart C., Reginster J.Y., Geerinck A., Locquet M., Bruyère O. (2017). Current Review of the SarQoL^®^: A Health-Related Quality of Life Questionnaire Specific to Sarcopenia. Expert Rev. Pharmacoeconomics Outcomes Res..

[23] Malmstrom T.K., Morley J.E. (2013). SARC-F: A Simple Questionnaire to Rapidly Diagnose Sarcopenia. J. Am. Med. Dir. Assoc..

[24] Cruz-Jentoft A.J., Bahat G., Bauer J., Boirie Y., Bruyère O., Cederholm T., Cooper C., Landi F., Rolland Y., Sayer A.A. (2019). Sarcopenia: Revised European Consensus on Definition and Diagnosis. Age Ageing.

[25] Guttikonda D., Smith A.L. (2021). Sarcopenia Assessment Techniques. Clin. Liver Dis..

[26] Holmes C.J., Racette S.B. (2021). The Utility of Body Composition Assessment in Nutrition and Clinical Practice: An Overview of Current Methodology. Nutrients.

[27] Cruz-Jentoft A.J., Baeyens J.P., Bauer J.M., Boirie Y., Cederholm T., Landi F., Martin F.C., Michel J.-P., Rolland Y., Schneider S.M. (2010). Sarcopenia: European Consensus on Definition and Diagnosis. Age Ageing.

[28] Saeki C., Takano K., Oikawa T., Aoki Y., Kanai T., Takakura K., Nakano M., Torisu Y., Sasaki N., Abo M. (2019). Comparative Assessment of Sarcopenia Using the JSH, AWGS, and EWGSOP2 Criteria and the Relationship between Sarcopenia, Osteoporosis, and Osteosarcopenia in Patients with Liver Cirrhosis. BMC Musculoskelet. Disord..

[29] Demonceau C., Voz B., Bruyère O., Reginster J.-Y., Beaudart C. (2024). Content Validity of SarQoL, a Quality of Life Questionnaire Specific to Sarcopenia. Aging Clin. Exp. Res..

[30] Dent E., Morley J.E., Cruz-Jentoft A.J., Arai H., Kritchevsky S.B., Guralnik J., Bauer J.M., Pahor M., Clark B.C., Cesari M. (2018). International Clinical Practice Guidelines for Sarcopenia (ICFSR): Screening, Diagnosis and Management. J. Nutr. Health Aging.

[31] Lu L., Mao L., Feng Y., Ainsworth B.E., Liu Y., Chen N. (2021). Effects of Different Exercise Training Modes on Muscle Strength and Physical Performance in Older People with Sarcopenia: A Systematic Review and Meta-Analysis. BMC Geriatr..

[32] Shen Y., Shi Q., Nong K., Li S., Yue J., Huang J., Dong B., Beauchamp M., Hao Q. (2023). Exercise for Sarcopenia in Older People: A Systematic Review and Network Meta-Analysis. J. Cachexia Sarcopenia Muscle.

[33] Chen N., He X., Feng Y., Ainsworth B.E., Liu Y. (2021). Effects of Resistance Training in Healthy Older People with Sarcopenia: A Systematic Review and Meta-Analysis of Randomized Controlled Trials. Eur. Rev. Aging Phys. Act..

[34] Rondanelli M., Cereda E., Klersy C., Faliva M.A., Peroni G., Nichetti M., Gasparri C., Iannello G., Spadaccini D., Infantino V. (2020). Improving Rehabilitation in Sarcopenia: A Randomized-Controlled Trial Utilizing a Muscle-Targeted Food for Special Medical Purposes. J. Cachexia Sarcopenia Muscle.

[35] Giallauria F., Cittadini A., Smart N.A., Vigorito C. (2016). Resistance Training and Sarcopenia. Monaldi Arch. Chest Dis..

[36] Amzolini A.M., Forțofoiu M.C., Barau Alhija A., Vladu I.M., Clenciu D., Mitrea A., Forțofoiu M., Matei D., Diaconu M., Tudor M.S. (2022). Triglyceride and Glucose Index as a Screening Tool for Nonalcoholic Liver Disease in Patients with Metabolic Syndrome. J. Clin. Med..

[37] Koleva I.B., Ioshinov B.R., Yoshinov R.D. (2017). Complex Analgesia (Infiltrations and Deep Oscillation) in Patients with Stump Pain and Phantom Pain After Lower Limb Amputation (Double-Blind Randomised Controlled Trial of Efficacy). J. Adv. Med. Med. Res..

[38] Locheva V., Todorov I., Panayotova-Ovcharova L. (2019). Therapy with Deep Oscillations—Principle, Biological Effects. Review Varna Med. Forum.

[39] Fistetto G., Iannitti T., Capone S., Torricelli F., Palmieri B. (2011). Deep Oscillation^®^: Esperienze Terapeutico-Riabilitative con un Nuovo Innovativo Strumento ad Azione Elettrostatica. Minerva Med..

[40] Rogan S., Taeymans J., Radlinger L., Naepflin S., Ruppen S., Bruelhart Y., Hilfiker R. (2017). Effects of Whole-Body Vibration on Postural Control in Elderly: An Update of a Systematic Review and Meta-Analysis. Arch. Gerontol. Geriatr..

[41] Weng S.E., Huang Y.W., Tseng Y.C., Peng H.R., Lai H.Y., Akishita M., Arai H., Hsiao F.Y., Chen L.K. (2025). The Evolving Landscape of Sarcopenia in Asia: A Systematic Review and Meta-Analysis Following the 2019 Asian Working Group for Sarcopenia (AWGS) Diagnostic Criteria. Arch. Gerontol. Geriatr..

[42] Bhasin S., Travison T.G., Manini T.M., Patel S., Pencina K.M., Fielding R.A., Magaziner J.M., Newman A.B., Kiel D.P., Cooper C. (2020). Sarcopenia Definition: The Position Statements of the Sarcopenia Definition and Outcomes Consortium. J. Am. Geriatr. Soc..

[43] Trăistaru R., Alexandru D.O., Kamal D., Kamal C.K., Rogoveanu O.C., Postolache P. (2018). Boswellia Derivates and Rehabilitation Program in Knee Osteoarthritis Patients. Rev. Chim..

[44] Trăistaru R., Alexandru D.O., Kamal D., Kamal C.K., Rogoveanu O. (2018). The Role of Herbal Extracts in Knee Osteoarthritis Females’ Rehabilitation. Farmacia.

[45] Kyle U.G., Bosaeus I., De Lorenzo A.D., Deurenberg P., Elia M., Gómez J.M., Heitmann B.L., Kent-Smith L., Melchior J.C., Pirlich M. (2004). Composition of the ESPEN Working Group. Bioelectrical impedance analysis—Part I: Review of principles and methods. Clin. Nutr..

[46] Janssen I., Heymsfield S.B., Wang Z., Ross R. (2000). Skeletal Muscle Mass and Distribution in 468 Men and Women Aged 18–88 Yr. J. Appl. Physiol..

[47] Christopher A., Kraft E., Olenick H., Kiesling R., Doty A. (2021). The reliability and validity of the Timed Up and Go as a clinical tool in individuals with and without disabilities across a lifespan: A systematic review. Disabil. Rehabil..

[48] Lauretani F., Russo C.R., Bandinelli S., Bartali B., Cavazzini C., Di Iorio A., Corsi A.M., Rantanen T., Guralnik J.M., Ferrucci L. (2003). Age-Associated Changes in Skeletal Muscles and Their Effect on Mobility: An Operational Diagnosis of Sarcopenia. J. Appl. Physiol..

[49] Guralnik J.M., Simonsick E.M., Ferrucci L., Glynn R.J., Berkman L.F., Blazer D.G., Scherr P.A., Wallace R.B. (1994). A Short Physical Performance Battery Assessing Lower Extremity Function: Association with Self-Reported Disability and Prediction of Mortality and Nursing Home Admission. J. Gerontol..

[50] Pavasini R., Guralnik J., Brown J.C., Di Bari M., Cesari M., Landi F., Vaes B., Legrand D., Verghese J., Wang C. (2016). Short Physical Performance Battery and All-Cause Mortality: Systematic Review and Meta-Analysis. BMC Med..

[51] Santamaría-Peláez M., González-Bernal J.J., Da Silva-González Á., Medina-Pascual E., Gentil-Gutiérrez A., Fernández-Solana J., Mielgo-Ayuso J., González-Santos J. (2023). Validity and Reliability of the Short Physical Performance Battery Tool in Institutionalized Spanish Older Adults. Nurs. Rep..

[52] SarQoL Questionnaire [Internet]. http://www.sarqol.org.

[53] Beaudart C., Demonceau C., Reginster J.Y., Locquet M., Cesari M., Cruz-Jentoft A.J., Bruyère O. (2023). Sarcopenia and Health-Related Quality of Life: A Systematic Review and Meta-Analysis. J. Cachexia Sarcopenia Muscle.

[54] Lee H., Kim J. (2024). Evaluating the SarQoL Questionnaire as a Screening Tool for Sarcopenia among Korean Older Adults. Healthcare.

[55] Erdogan T., Eris S., Avci S., Oren M.M., Kucukdagli P., Kilic C., Beaudart C., Bruyere O., Karan M.A., Bahat G. (2021). Sarcopenia Quality-of-Life Questionnaire (SarQoL)^®^: Translation, Cross-Cultural Adaptation and Validation in Turkish. Aging Clin. Exp. Res..

[56] Montero-Errasquín B., Vaquero-Pinto N., Sánchez-Cadenas V., Geerinck A., Sánchez-García E., Mateos-Nozal J., Ribera-Casado J.M., Cruz-Jentoft A.J. (2022). Spanish Translation, Cultural Adaptation and Validation of the SarQoL^®^: A Specific Health-Related Quality of Life Questionnaire for Sarcopenia. BMC Musculoskelet. Disord..

[57] Reginster J.Y., Beaudart C., Al-Daghri N., Avouac B., Bauer J., Bere N., Bruyère O., Cerreta F., Cesari M., Rosa M.M. (2021). Update on the ESCEO Recommendation for the Conduct of Clinical Trials for Drugs Aiming at the Treatment of Sarcopenia in Older Adults. Aging Clin. Exp. Res..

[58] Wu S., Ning H.T., Xiao S.M., Hu M.-Y., Wu X.-Y., Deng H.-W., Feng H. (2020). Effects of Vibration Therapy on Muscle Mass, Muscle Strength and Physical Function in Older Adults with Sarcopenia: A Systematic Review and Meta-Analysis. Eur. Rev. Aging Phys. Act..

[59] Matei D., Marcu I.R., Pătru G.L., Pătru L., Bighea A.C., Pătru S. (2019). A Case of Giant Cell Tumor of the Tendon Sheath in an Elderly Patient: Diagnostic Difficulties and Therapeutic Options. Rom. J. Morphol. Embryol..

[60] Popescu C., Matei D., Amzolini A.M., Trăistaru M.R. (2024). Inflammation and Physical Performance in Overweight and Obese Schoolchildren. Life.

[61] Matei D., Trăistaru R., Pădureanu V., Avramescu T.E., Neagoe D., Genunche A., Amzolini A. (2024). The Efficiency of Kinesiotherapy versus Physical Modalities on Pain and Other Common Complaints in Fibromyalgia. Life.

[62] Soriano J.M., Fernández-Garrido J.J. (2022). SarQoL Questionnaire in Community-Dwelling Older Adults under EWGSOP2 Sarcopenia Diagnosis Algorithm: A New Screening Method?. Int. J. Environ. Res. Public Health.

[63] Drăgoi D., Popescu R., Trăistaru R., Matei D., Buzatu A.M., Ionovici N., Grecu D. (2010). A Multidisciplinary Approach in Patients with Femoral Neck Fracture on an Osteoporotic Basis. Rom. J. Morphol. Embryol..

[64] Zhao H., Cheng R., Song G., Teng J., Shen S., Fu X., Yan Y., Liu C. (2022). The Effect of Resistance Training on the Rehabilitation of Elderly Patients with Sarcopenia: A Meta-Analysis. Int. J. Environ. Res. Public Health.

[65] Cesari M., Landi F., Calvani R., Cherubini A., Di Bari M., Kortebein P., Del Signore S., Regis Le Lain S., Vellas B., Pahor M. (2017). Rationale for a Preliminary Operational Definition of Physical Frailty and Sarcopenia in the SPRINTT Trial. Aging Clin. Exp. Res..

